# Prior Cardiovascular Disease and Incident Diabetic Retinopathy in Newly Diagnosed Type 2 Diabetes Mellitus

**DOI:** 10.3390/jcm15218544

**Published:** 2026-11-03

**Authors:** Jung Soo Yoon, Jiyeong Kim, Lei Liu, Dongjin Nam, Sunjin Hwang

**Affiliations:** 1 Department of Plastic and Reconstructive Surgery, Myongji Hospital, Myongji Medical Foundation, Goyang 10475, Republic of Korea; yoonjsps@mjh.or.kr; 2 Department of Pre-Medicine, Hanyang University College of Medicine, Seoul 04763, Republic of Korea; kimzi@hanyang.ac.kr; 3 Biostatistics Laboratory, Medical Research Collaborating Center, Hanyang University, Seoul 04763, Republic of Korea; 4 Department of Ophthalmology, Guangdong Eye Institute, Guangdong Provincial People’s Hospital (Guangdong Academy of Medical Sciences), Southern Medical University, Guangzhou 510080, China; liulei@gdph.org.cn; 5 Guangdong Provincial Key Laboratory of Artificial Intelligence in Medical Image Analysis and Application, Guangzhou 510080, China; 6 Department of Internal Medicine, Graduate School, Yonsei University College of Medicine, Seoul 03722, Republic of Korea; jayden.nam@mediwhale.com; 7 Mediwhale Inc., Seoul 06049, Republic of Korea; 8 Department of Ophthalmology, Hanyang University Guri Hospital, Guri 11923, Republic of Korea; 9 Department of Ophthalmology, Hanyang University College of Medicine, Seoul 04763, Republic of Korea

**Keywords:** cardiovascular disease, diabetic retinopathy, type 2 diabetes

## Abstract

**Background/Objectives:** To determine whether prior cardiovascular disease (CVD) independently predicts diabetic retinopathy (DR) in adults with newly diagnosed type 2 diabetes mellitus (T2DM). **Methods:** A retrospective nationwide population-based cohort study was conducted using the Korean National Health Insurance Service database. Adults aged ≥40 years with newly diagnosed T2DM were identified after a 7-year washout period. After 1:1 propensity score matching on age and sex, the analytical cohort comprised 38,812 patients per group. **Results:** After full covariate adjustment, prior CVD was not independently associated with any DR (HR, 1.01; 95% confidence interval [CI], 0.99–1.04; *p* = 0.262), NPDR (HR, 1.01; 95% CI, 0.98–1.03; *p* = 0.605), PDR (HR, 1.01; 95% CI, 0.95–1.06; *p* = 0.835), or VTDR (HR, 1.00; 95% CI, 0.95–1.06; *p* = 0.893). Fine–Gray models, accounting for higher all-cause mortality in the prior CVD group after matching (25.2% vs. 22.1%), revealed slightly lower sHR for any DR (sHR, 0.97; 95% CI, 0.95–0.99; *p* = 0.014) and NPDR (sHR, 0.97; 95% CI, 0.94–0.99; *p* = 0.005). **Conclusions:** Prior CVD was not independently associated with incident DR. The lower sHRs may partly reflect competing mortality and should not be interpreted as DR protection. Overall, CVD history was not a criterion for intensified DR screening.

## 1. Introduction

Diabetic retinopathy (DR) is the leading cause of preventable blindness among working-age adults worldwide, and remains one of the most frequent and vision-threatening complications of type 2 diabetes mellitus (T2DM) [1,2]. Globally, DR affects approximately one-third of all individuals with diabetes, with vision-threatening DR present in approximately 10% of the diabetic population. As the worldwide prevalence of diabetes continues to rise, the estimated number of adults living with DR is projected to increase from 103 million in 2020 to 160 million by 2045, placing an escalating burden on patients, healthcare systems, and economies [2]. Early identification of high-risk individuals at the time of diabetes diagnosis is critical because timely ophthalmological intervention can preserve vision and prevent irreversible sight loss.

Cardiovascular disease (CVD) and DR are not merely concurrent manifestations of diabetes but share a common pathophysiological substrate rooted in systemic vascular dysfunction [3,4]. Chronic hyperglycemia drives endothelial injury, oxidative stress, low-grade inflammation, and basement membrane thickening in both the retinal microcirculation and the coronary and peripheral macrovasculature through overlapping molecular mechanisms including activation of advanced glycation end-products, the polyol pathway, and protein kinase C [1,5]. Epidemiological evidence reinforces this biological continuum: a meta-analysis of 20 prospective studies demonstrated that the presence of DR in patients with T2DM was associated with more than a two-fold increase in the risk of cardiovascular events and all-cause mortality, independent of classical risk factors [6]. From this shared biology, it is plausible to hypothesize that patients with pre-existing CVD at the time of T2DM diagnosis may be at higher risk of DR development or faster progression to proliferative diabetic retinopathy (PDR) or vision-threatening diabetic retinopathy (VTDR).

Several critical methodological gaps in clinical studies have precluded definitive conclusions. First, most studies examining the CVD–DR relationship have focused on the reverse direction or have been conducted in populations with prevalent, long-standing diabetes, making it difficult to isolate the effect of preexisting CVD on retinal outcomes from the effect of cumulative diabetes duration [7,8]. Studies specifically examining whether prior CVD predicts incident DR in newly diagnosed T2DM are limited [9,10]. Second, patients with established CVD have substantially higher mortality than those without; failure to account for death as a competing event inflates apparent cause-specific hazard differences [11,12,13].

To address these gaps, we aimed (1) to determine whether prior CVD independently predicts the incidence and 5- and 10-year severity distribution of DR and (2) to quantify the rate and speed of NPDR to PDR (or VTDR) progression by prior CVD status.

## 2. Materials and Methods

### 2.1. Ethics Statement

This study was approved by the Institutional Review Board of the Hanyang University Guri Hospital (IRB number: GURI 2025-08-007). The Institutional Review Board waived the requirement for informed consent because de-identified information was used in the analyses.

### 2.2. Study Design and Datasets

We performed a retrospective population-based cohort study using nationwide claims data from the Korean National Health Insurance Service (KNHIS), a mandatory universal health insurance system that covers the entire South Korean population and captures comprehensive information on healthcare utilization [14,15,16]. The study population comprised adults aged ≥40 years with a first-ever diagnosis of T2DM between 1 January 2009 and 31 December 2012. Newly diagnosed T2DM was defined as having an International Classification of Diseases 10th revision (ICD-10) diagnosis code for T2DM (E11, except E10, 12, 13, and 14) during 2009–2012, with no record of any diabetes diagnosis or diabetes-related complications in the preceding years between 2002 and 2008. To minimize reverse causality from latent preexisting diabetes, participants who developed any DR or died within 1 year of their T2DM diagnosis were also excluded. Because follow-up began 1 year after T2DM diagnosis, the study population constituted a 1-year landmark cohort of patients who survived without DR during the first year after diagnosis. The overall exclusion process is illustrated in Supplementary Figure S1.

### 2.3. Exposure Definition

The primary exposure was prior CVD, defined as any diagnosis of ischemic heart disease (KCD-8 I20–I25), heart failure (I50), stroke (I60–I69), or peripheral artery disease (I70, I73, I79.2) recorded during the washout period of 2002–2008. Patients fulfilling this definition comprised the prior-CVD group, and all the remaining patients constituted the no-prior-CVD group. Propensity score matching (PSM) was used to obtain a balanced comparison cohort.

### 2.4. Outcome Definition

Primary outcomes were any DR, NPDR, PDR, and VTDR. NPDR was identified by the presence of at least one of the following KCD codes: E11.31, E11.33, H35.0, or H36.0. PDR was defined as H45.0, H33.4, H33.5, H35.2, H43.1, E11.32, or H35.80, or NPDR codes in combination with retinal laser photocoagulation (S5160). VTDR was identified by the presence of at least one of the following KCD codes: H35.80, H43.1, H45.0, H33.4, H33.5, or H35.2, or by receipt of procedures (S4880, S4900, S5121, S5122, S5130, or S5164). DR severity was classified hierarchically, with VTDR superseding PDR and NPDR, and PDR superseding NPDR, when codes for multiple severity categories co-occurred. The index date for follow-up was 1 year after T2DM diagnosis; participants were followed up until the first occurrence of the relevant DR outcome, death, or 31 December 2024, whichever occurred first. For the NPDR-to-PDR (or VTDR) progression analysis, the index date was the date of the first NPDR diagnosis, and follow-up was continued until PDR diagnosis, death, or censoring.

### 2.5. Covariate Definition

Baseline covariates were ascertained from claims records and health examination data at or near the time of T2DM diagnosis and included: age at diagnosis (categorized as 40–49, 50–59, 60–69, 70–79, and ≥80 years), sex, smoking status (never, ex-smoker, or current smoker), alcohol consumption (none/light [<3 days/week] and moderate/heavy [≥3 days/week]), insurance type, region of residence (Seoul; Gyeonggi/metropolitan cities; other areas), statin use intensity (none, group 1 [low], group 2 [moderate], and group 3 [high], based on type and cumulative prescription days within the first year), pre-index ophthalmological visit (≥1 visit in the year before T2DM diagnosis), body mass index (BMI; <23.0, 23.0–24.9, or ≥25.0 kg/m^2^), and fasting blood glucose (FBG; <130, 130–179, or ≥180 mg/dL). Comorbidities defined from the full claims history (≥2 diagnoses at any time point) are listed in Supplementary Table S1. Data on total cholesterol, triglycerides, HDL, and LDL levels and blood pressure were obtained from the health examination database.

All-cause mortality was assessed using linked death records from the KNHIS. The follow-up for mortality was completed for all individuals until 31 December 2024.

### 2.6. Statistical Analysis

Baseline characteristics are described as means ± standard deviation (SD) for continuous variables and as frequencies (proportions) for categorical variables; between-group differences were assessed by Student’s *t*-test or chi-square test. To balance age and sex between the prior-CVD and no-prior-CVD groups, we applied a 1:1 PSM using the nearest-neighbor algorithm, with propensity scores estimated by logistic regression including age and sex. Covariate balance was evaluated using standardized mean differences (SMDs); SMD < 0.1 was considered adequate. The remaining covariates were not included in the matching procedure; instead, key covariates were adjusted for in the multivariable Cox and Fine–Gray models.

**Figure 1:**
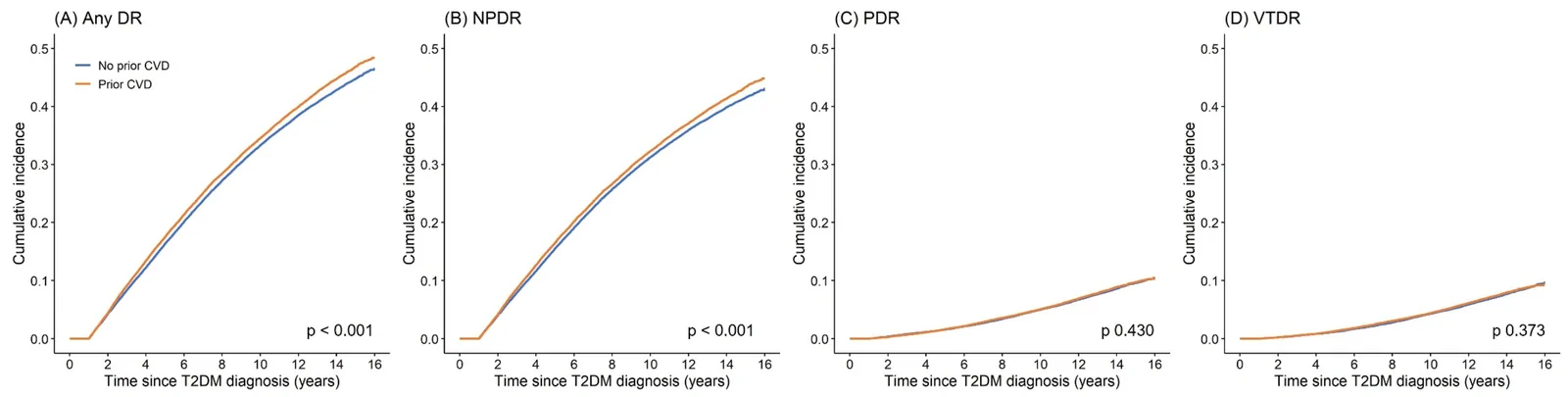
Kaplan–Meier cumulative incidence curves for DR outcomes by prior CVD status. Cumulative incidences of (**A**) any DR, (**B**) NPDR, (**C**) PDR, and (**D**) VTDR in the propensity score–matched cohort (n = 38,812 per group) for up to 16 years of follow-up after T2DM diagnosis. The orange lines represent the prior-CVD group, and the blue lines represent the no-prior-CVD group. *p*-values were obtained using log-rank tests. Note that these Kaplan–Meier curves do not account for death as a competing event. Abbreviations: CVD, cardiovascular disease; DR, diabetic retinopathy; NPDR, non-proliferative diabetic retinopathy; PDR, proliferative diabetic retinopathy; VTDR, vision-threatening diabetic retinopathy; T2DM, type 2 diabetes mellitus.

Cox proportional hazards regression was used to estimate hazard ratios (HRs) and 95% confidence intervals (CIs) for the association between prior CVD and incident DR using four sequential models: crude (unadjusted), Model 1 (age and sex), Model 2 (Model 1 + smoking, alcohol, insurance, residence, hypertension, dyslipidemia, BMI, and FBG), and Model 3 (Model 2 + pre-index ophthalmological visit). As patients with prior CVD experienced higher all-cause mortality (25.2% vs. 22.1%, *p* < 0.001), death constituted a competing event that prevented DR observation. Therefore, we additionally estimated sub-distribution HRs (sHRs) using the Fine–Gray proportional sub-distribution hazards model, with death treated as the competing event, under the same four covariate models. Cumulative incidence functions (CIFs) for DR outcomes were estimated, accounting for competing mortality, and are displayed in Supplementary Figure S2A–D alongside cause-specific Kaplan–Meier curves (Figure 1). Gray’s test was used to compare the CIFs between groups.

The 5- and 10-year maximum observed DR severity distributions were compared between groups using multinomial logistic regression, yielding adjusted relative risk ratios (RRRs) with 95% CIs, with the No DR category as a reference. For the progression analysis, time from the first NPDR diagnosis to the first PDR diagnosis was summarized by median (IQR) and mean ± SD, with between-group comparisons using the Wilcoxon rank-sum test. All analyses were performed using the SAS Enterprise Guide v.7.1 (SAS Institute Inc., Cary, NC, USA) and R v.4.5 (R Foundation for Statistical Computing, Vienna, Austria). A two-sided *p* < 0.05 was considered statistically significant.

### 2.7. Sensitivity Analysis

To assess the robustness of the primary findings, two pre-specified sensitivity analyses were conducted using both cause-specific Cox and Fine–Gray models. First, to reduce the potential reverse causality from very early DR, which may reflect longstanding undiagnosed diabetes, we extended the exclusion window from 1 to 2 years after T2DM diagnosis (lag-2 analysis). Second, to evaluate the influence of differential healthcare utilization, we restricted the analyses to patients who had at least one ophthalmological outpatient visit within 1 year of T2DM diagnosis (early exam cohort).

## 3. Results

### 3.1. Cohort Characteristics

The inclusion and exclusion processes are summarized in Supplementary Figure S1. After 1:1 PSM, the final analytical cohort comprised 38,812 patients with prior CVD and 38,812 without prior CVD.

Before PSM, the prior-CVD group was substantially older (SMD 0.823; mean age 60–69 years was the most common [31.7%] vs. 50–59 years in the no-CVD group [38.2%]), included more women (53.1% vs. 36.5%), had higher rates of never-smoking (65.7% vs. 50.5%), and had substantially more comorbidities: hypertension (91.0% vs. 72.4%), heart failure (25.6% vs. 8.1%), and COPD (18.1% vs. 8.1%). The prior-CVD group was more likely to have had pre-index (36.6% vs. 24.8%) and post-index (39.5% vs. 29.3%) ophthalmological visits. The all-cause mortality in the prior-CVD group was more than twice as high as that in the no-CVD group (26.1% vs. 12.6%). After PSM, age and sex were well balanced between the groups (Table 1, Supplementary Table S2).

### 3.2. Association of Prior CVD with Incident DR

During follow-up, 14,822 DR events (NPDR, PDR, or VTDR) occurred in the no-CVD group (incidence rate [IR] 39.09 per 1000 person-years [PY]) and 15,160 in the prior-CVD group (IR 41.34 per 1000 PY). IRs were similar between the two groups for both PDR (6.15 vs. 6.23 per 1000 person-years) and VTDR (5.45 vs. 5.54 per 1000 person-years) (Table 2).

In crude Cox models, prior CVD was marginally associated with any DR and NPDR but not PDR and VTDR. However, these associations attenuated after sequential adjustment and became non-significant in the fully adjusted model for all DR outcomes. Kaplan–Meier curves showed only minimal separation between groups for any DR and NPDR, while PDR and VTDR incidence was similar throughout follow-up (Figure 1).

Fine–Gray competing-risk models, accounting for the substantially higher mortality in the prior-CVD group, showed a notably different pattern. In the fully adjusted Model 3, prior CVD was associated with a significantly lower sHR for any DR (sHR 0.97, 95% CI 0.95–0.99; *p* = 0.014) and NPDR (sHR 0.97, 95% CI 0.94–0.99; *p* = 0.005), and a borderline non-significant lower sHR for PDR (sHR 0.96, 95% CI 0.91–1.01; *p* = 0.108) and VTDR (sHR 0.95, 95% CI 0.90–1.01; *p* = 0.089). These findings suggest that the lower sHRs in the prior-CVD group may partly reflect competing mortality rather than retinal protection (Supplementary Figure S2).

### 3.3. Five- and Ten- Year Severity Distribution of DR

At 5 years, DR stage distributions were similar between groups, with no significant associations for NPDR, PDR, or VTDR after adjustment. By 10 years, adjusted multinomial regression showed slightly lower relative risks of NPDR (RRR 0.95, 95% CI 0.92–0.99), PDR (RRR 0.97, 95% CI 0.85–1.11), and VTDR (RRR 0.94, 95% CI 0.87–1.01) in the prior-CVD group, consistent with competing-risk effects from higher mortality rather than retinal protection (Table 3, Supplementary Figure S3).

### 3.4. NPDR to PDR (Or VTDR) Progression

Among patients who progressed from NPDR to PDR (or VTDR) (n = 2727), progression was slightly slower in the prior-CVD group than in the no-CVD group (median 40.45 vs. 38.70 months; *p* = 0.041). However, prior CVD was not associated with accelerated NPDR-to-PDR (or VTDR) progression overall (Supplementary Table S3, Supplementary Figure S4).

### 3.5. Sensitivity Analyses

Sensitivity analyses showed findings consistent with the primary analysis. In both the lag-2 and early exam analyses, prior CVD was not associated with increased risks of any DR, NPDR, PDR, or VTDR in either cause-specific Cox or Fine–Gray models, supporting the robustness of the main results (Supplementary Table S4).

## 4. Discussion

In this nationwide population-based cohort study, prior CVD was not independently associated with incident DR after full covariate adjustment. When death was appropriately modeled as a competing event using Fine–Gray regression, prior CVD was associated with a significantly lower sHR for any DR and NPDR. NPDR-to-PDR (or VTDR) progression was slower in the prior-CVD group. Together, these findings challenge the intuitive assumption that the macrovascular disease burden accelerates the retinal disease trajectory in newly diagnosed patients with T2DM.

The null association between prior CVD and DR incidence observed after full adjustment is consistent with emerging evidence that, although DR and CVD share a common systemic vascular substrate, their independent trajectories from the time of T2DM diagnosis may diverge [4]. Cross-sectional studies have reported that established CVD is associated only with PDR but not NPDR severity [17]. Furthermore, biomarker research suggests that distinct molecular pathways contribute to the two complication types [18]. Longitudinal data from the Japan Diabetes Complications Study, whose any DR incidence rate of 38.3 per 1000 PY closely mirrors that of the present study of approximately 40 per 1000 PY, identified glycated hemoglobin as the sole independent predictor of both DR incidence and progression, with no contribution from macrovascular risk markers [19]. Our results confirm this hierarchy in a much larger cohort and extend it to explicitly include prior CVD as a tested, non-significant predictor. The analogous finding in the KNHIS dataset [20], whereby atrial fibrillation, another cardiovascular burden marker, similarly did not predict incident DR in the same cohort window, further supports the conclusion that macrovascular-related burden does not constitute an independent retinal risk factor early in the course of T2DM.

The discordance between cause-specific HRs and Fine–Gray sHRs reflects the different questions addressed by these models. Cause-specific Cox models estimate the rate of DR among patients who remain alive and DR-free, whereas Fine–Gray models assess associations with cumulative DR incidence while accounting for death as a competing event [11,12]. The higher all-cause mortality in the prior-CVD group (25.2% vs. 22.1%) may partly explain why sHRs were below 1 despite cause-specific HRs near 1, as death limits the opportunity for subsequent DR diagnosis [13]. Accordingly, the lower sHRs should be interpreted in the context of competing mortality and should not be taken as evidence that prior CVD protects against DR [21]. Competing mortality should thus be considered when comparing cumulative DR incidence between patients with and without prior CVD.

The slower NPDR-to-PDR (or VTDR) progression in the prior-CVD group warrants cautious interpretation, as competing mortality alone is unlikely to fully account for this difference. Although preferential early death among patients with the most rapidly progressing CVD may have plausibly selectively removed the highest-risk individuals from long-term follow-up [6,13], the modest mortality difference between the groups after PSM (25.2% vs. 22.1%) and the relatively small absolute difference in progression timing argue against this as the sole explanation. An additional contributing factor may be differential ascertainment; patients in the prior CVD group attended ophthalmological visits more frequently, potentially enabling earlier NPDR detection at a less advanced stage, which could, in turn, produce an artificially longer observed time to PDR (or VTDR). Furthermore, the larger absolute number of NPDR-to-PDR (or VTDR) progressors in the non-CVD group suggests that factors beyond competing mortality, including differences in systemic risk factor management and medication intensity, may contribute to this pattern. Korean cohort data have shown that renal function decline, which is closely linked to systemic microvascular injury rather than macrovascular disease, is the dominant predictor of NPDR-to-PDR (or VTDR) progression [22,23], and our results suggest that a history of macrovascular CVD adds little to this prediction. Altogether, the slower progression observed in the prior-CVD group likely reflects a composite of competing mortality, differential screening access, and potentially more aggressive vascular risk factor management in this population, rather than any direct DR-protective effect of prior CVD.

An additional possible confounding scenario should be considered. Patients who survived prior CVD episodes may have been more incentivized to control their newly diagnosed diabetes than those without prior CVD. This motivation could have been reinforced by clinical staff, who naturally assume that previous systemic vascular dysregulation confers a higher chance of DR progression at a given HbA1c level, thereby prompting more intensive metabolic management. Such differential care could have offset any excess retinal risk associated with prior CVD, contributing to the null association observed in this study. As data on HbA1c trajectories, treatment intensification, and adherence were unavailable in our dataset, we could not directly test this hypothesis. Whether heightened patient motivation and intensified clinical vigilance after a cardiovascular event offset the excess risk of DR development and progression warrants further study.

The principal strengths of this study were its nationwide scale, a comprehensive population, and methodological rigor. With 77,624 age- and sex-matched participants, a 7-year washout period, up to 16 years of follow-up, and explicit application of Fine–Gray competing-risks regression, this study provides large-scale evidence on the CVD–DR relationship in patients with newly diagnosed T2DM. The use of the KNHIS database minimized selection bias and enabled complete outcome assessment across all healthcare settings. The pre-specification of sensitivity analyses and consistent null results across these analyses strengthen the causal inference.

This study has certain limitations that warrant consideration. First, prior CVD was ascertained during the washout period (2002–2008) to apply a uniform ascertainment period and to limit prior CVD to events recorded in the absence of diabetes. Consequently, patients first diagnosed with CVD between 2009 and their T2DM diagnosis were classified into the non-CVD group, which may have biased the results toward the null. Second, DR ascertainment relied on diagnostic and procedure codes rather than standardized retinal photography. Claim-based DR detection is inherently visit-dependent, and mild NPDR managed without formal documentation may be under-ascertained. Third, HbA1c data were unavailable; because glycemic control is the dominant determinant of DR [24], residual confounding by glycemic exposure cannot be excluded, and the null association should be interpreted with caution. Better glycemic control after diagnosis among patients with prior CVD, as discussed above, could have masked a modest excess risk. Fourth, matching was performed on age and sex only, and residual imbalances in comorbidity and healthcare-utilization variables, although adjusted for, could still have influenced DR ascertainment. Fifth, the 1-year landmark design excluded patients who developed DR or died within the first year after T2DM diagnosis (n = 29,802). Because these patients may have had longstanding undiagnosed diabetes or severe comorbidities, this exclusion may have introduced selection bias, and our findings may not be generalizable to patients at high early risk. Sixth, our cohort was restricted to Korean patients, which limits generalizability of the findings to other ethnic populations in which DR risk profiles and CVD prevalence differ [2]. Seventh, the analysis of progression from NPDR to PDR (or VTDR) was descriptive and restricted to patients who progressed, without accounting for death as a competing event; thus, it does not reflect progression risk in the full NPDR cohort.

## 5. Conclusions

In this large nationwide cohort of patients with newly diagnosed T2DM, prior CVD was not independently associated with DR incidence; the modest crude associations may be partly explained by differences in ophthalmological surveillance and competing mortality. Our findings do not support prior CVD as an additional criterion for intensified DR screening. This statement applies to patients who already meet standard DR-screening indications and does not imply that patients with prior CVD can forgo screening; they should continue to undergo guideline-recommended DR screening.

## Figures and Tables

**Table 1:** Baseline demographics of propensity score–matched cohort.

Variable	Without Prior CVD (n = 38,812)	With Prior CVD (n = 38,812)	SMD	*p* Value
Age at diagnosis, years			0.008	0.333
40–49	3936 (10.1%)	3936 (10.1%)		
50–59	11,282 (29.1%)	11,282 (29.1%)		
60–69	12,660 (32.6%)	12,558 (32.4%)		
70–79	9192 (23.7%)	9120 (23.5%)		
≥80	1742 (4.5%)	1916 (4.9%)		
Male sex	18,614 (48.0%)	18,590 (47.9%)	0.001	0.863
Smoking			0.041	<0.001
Never smoker	24,931 (64.2%)	25,250 (65.1%)		
Ex-smoker	6391 (16.5%)	6683 (17.2%)		
Current smoker	7490 (19.3%)	6879 (17.7%)		
Alcohol consumption			0.033	<0.001
None/light (<3 days/wk)	33,259 (85.7%)	33,703 (86.8%)		
Moderate/heavy (≥3 days/wk)	5553 (14.3%)	5109 (13.2%)		
Medical aid (low SEP)	674 (1.7%)	1347 (3.5%)	0.109	<0.001
Region of residence			0.107	<0.001
Seoul	6657 (17.2%)	5540 (14.3%)		
Gyeonggi/metropolitan	18,019 (46.4%)	17,110 (44.1%)		
Other areas	14,136 (36.4%)	16,162 (41.6%)		
Statin intensity			0.147	<0.001
None	9680 (24.9%)	7321 (18.9%)		
Group 1 (low)	259 (0.7%)	231 (0.6%)		
Group 2 (moderate)	25,971 (66.9%)	27,226 (70.2%)		
Group 3 (high)	2902 (7.5%)	4034 (10.4%)		
Pre-index ophtho visit (≥1, pre-T2DM yr)	11,478 (29.6%)	14,111 (36.4%)	0.145	<0.001
Post-index ophtho visit (≥1, post-T2DM yr)	13,264 (34.2%)	15,230 (39.2%)	0.105	<0.001
Comorbidities				
Hypertension	30,056 (77.4%)	35,306 (91.0%)	0.377	<0.001
Dyslipidemia	33,421 (86.1%)	35,531 (91.6%)	0.173	<0.001
Liver disease	31,497 (81.2%)	33,795 (87.1%)	0.163	<0.001
COPD	4913 (12.7%)	6940 (17.9%)	0.146	<0.001
Health examination data				
BMI category			0.150	<0.001
<23.0 kg/m^2^	11,453 (29.5%)	9152 (23.6%)		
23.0–24.9 kg/m^2^	9638 (24.8%)	9031 (23.3%)		
≥25.0 kg/m^2^	17,721 (45.7%)	20,629 (53.2%)		
Fasting blood glucose			0.187	<0.001
<130 mg/dL	24,453 (63.0%)	27,843 (71.7%)		
130–179 mg/dL	10,578 (27.3%)	8650 (22.3%)		
≥180 mg/dL	3781 (9.7%)	2319 (6.0%)		
Total cholesterol, mg/dL	197.00 ± 47.53	191.81 ± 47.90	0.109	<0.001
Triglycerides, mg/dL	161.49 ± 112.89	163.66 ± 117.58	0.019	0.009
HDL cholesterol, mg/dL	52.06 ± 20.89	51.30 ± 23.96	0.034	<0.001
LDL cholesterol, mg/dL	111.23 ± 46.40	107.97 ± 49.82	0.104	<0.001

Abbreviations: BMI, body mass index; COPD, chronic obstructive pulmonary disease; CVD, cardiovascular disease; HDL, high-density lipoprotein; LDL, low-density lipoprotein; SEP, socioeconomic position; SMD, standardized mean difference; T2DM, type 2 diabetes mellitus. Data are presented as n (%) for categorical variables and mean ± SD for continuous variables. Propensity scores were estimated using age and sex only; other covariates were not included in the matching procedure. SMD < 0.1 indicates adequate balance between groups.

**Table 2:** Association of prior CVD with incident diabetic retinopathy.

Outcome	No CVD Events/PY	No CVD IR/1000PY	Prior CVD Events/PY	Prior CVD IR/1000PY	Model	HR (95% CI)	*p*-Value	Fine-Gray sHR (95% CI)	*p*-Value
Any DR	14,822/379,158	39.09	15,160/366,733	41.34	Crude	1.06 (1.04–1.08)	<0.0001	1.03 (1.01–1.06)	0.004
					Model 1	1.06 (1.04–1.09)	<0.0001	1.04 (1.02–1.06)	0.001
					Model 2	1.03 (1.01–1.06)	0.011	0.99 (0.97–1.01)	0.293
					Model 3	1.01 (0.99–1.04)	0.262	0.97 (0.95–0.99)	0.014
NPDR	13,792/385,296	35.80	14,056/373,477	37.64	Crude	1.05 (1.03–1.08)	<0.0001	1.03 (1.00–1.05)	0.021
					Model 1	1.05 (1.03–1.08)	<0.0001	1.03 (1.01–1.06)	0.006
					Model 2	1.02 (1.00–1.05)	0.067	0.98 (0.96–1.01)	0.136
					Model 3	1.01 (0.98–1.03)	0.605	0.97 (0.94–0.99)	0.005
PDR	2890/469,703	6.15	2862/459,191	6.23	Crude	1.02 (0.97–1.08)	0.430	0.99 (0.94–1.05)	0.775
					Model 1	1.02 (0.97–1.08)	0.423	1.00 (0.95–1.05)	0.881
					Model 2	1.02 (0.97–1.08)	0.465	0.97 (0.92–1.02)	0.279
					Model 3	1.01 (0.95–1.06)	0.835	0.96 (0.91–1.01)	0.108
VTDR	2572/472,064	5.45	2553/461,208	5.54	Crude	1.03 (0.97–1.08)	0.373	1.00 (0.94–1.05)	0.882
					Model 1	1.03 (0.97–1.09)	0.339	1.00 (0.95–1.06)	0.988
					Model 2	1.02 (0.96–1.08)	0.518	0.97 (0.91–1.02)	0.237
					Model 3	1.00 (0.95–1.06)	0.893	0.95 (0.90–1.01)	0.089

Model definitions: Crude, unadjusted; Model 1, adjusted for age and sex; Model 2, Model 1 plus smoking, alcohol consumption, insurance type, region of residence, hypertension, dyslipidemia, BMI, and fasting blood glucose; Model 3, Model 2 plus pre-index ophthalmological outpatient visits within 1 year before T2DM diagnosis. Fine–Gray sHRs were estimated using all-cause death as a competing event under the same covariate adjustment models. Abbreviations: BMI, body mass index; CVD, cardiovascular disease; DR, diabetic retinopathy; HR, hazard ratio; IR, incidence rate; NPDR, non-proliferative diabetic retinopathy; PDR, proliferative diabetic retinopathy; VTDR, vision-threatening diabetic retinopathy; PY, person-years; sHR, subdistribution hazard ratio; T2DM, type 2 diabetes mellitus.

**Table 3:** Adjusted relative risk ratios for 5- and 10-year distribution of diabetic retinopathy according to prior CVD status.

Final DR Status	5-Year Follow-Up			10-Year Follow-Up		
	No CVD, n (%)	Prior CVD, n (%)	Adjusted RRR (95% CI)	No CVD, n (%)	Prior CVD, n (%)	Adjusted RRR (95% CI)
No DR (reference)	32,709 (84.3%)	32,321 (83.3%)	1	26,908 (69.3%)	26,630 (68.6%)	1
NPDR	5434 (14.0%)	5802 (15.0%)	1.01 (0.97–1.06)	9961 (25.7%)	10,216 (26.3%)	0.95 (0.92–0.99) *
PDR	227 (0.6%)	206 (0.5%)	0.85 (0.70–1.04)	441 (1.1%)	450 (1.2%)	0.97 (0.85–1.11) *
VTDR	442 (1.1%)	483 (1.2%)	1.00 (0.88–1.15)	1502 (3.9%)	1516 (3.9%)	0.94 (0.87–1.01) *

The RRRs were estimated using multinomial logistic regression with No DR as the reference category, adjusted for age, sex, smoking, alcohol consumption, insurance type, region of residence, hypertension, dyslipidemia, BMI, fasting blood glucose level, and pre-index ophthalmological visit. * *p* < 0.05. Note that RRRs <1 at 10 years for prior CVD versus no CVD reflect competitive suppression by higher mortality in the prior CVD group (see text). Abbreviations: BMI, body mass index; CVD, cardiovascular disease; DR, diabetic retinopathy; NPDR, non-proliferative diabetic retinopathy; PDR, proliferative diabetic retinopathy; VTDR, vision-threatening diabetic retinopathy; RRR, relative risk ratio.

## Data Availability

The data used in this study were provided by the National Health Insurance Service (NHIS) of Korea under a data use agreement and are not publicly available due to restrictions imposed by the data provider to protect patient privacy. Researchers who wish to access the data may apply directly to the NHIS through its official data request system (https://nhiss.nhis.or.kr).

## Supplementary Materials

The following supporting information can be downloaded at https://www.mdpi.com/article/10.3390/jcm15218544/s1: Figure S1: Flowchart of study participants selection; Figure S2: Fine–Gray competing-risks cumulative incidence functions for DR outcomes; Figure S3: Five- and ten-year maximum DR severity distribution by prior CVD status; Figure S4: Violin plot of time from NPDR to PDR (or VTDR) onset by prior CVD status; Table S1: Detailed definitions of comorbidities and diabetes-related microvascular complications other than diabetic retinopathy; Table S2: Clinical outcomes during follow-up according to prior CVD status; Table S3: Time from NPDR to PDR (or VTDR) progression stratified by prior CVD status (N = 2727); Table S4: Sensitivity Analyses for the Association Between Prior CVD and Incident DR.

## Abbreviations

The following abbreviations are used in this manuscript:

BMI	Body mass index
CI	Confidence interval
CIF	Cumulative incidence function
COPD	Chronic obstructive pulmonary disease
CVD	Cardiovascular disease
DR	Diabetic retinopathy
ESRD	End-stage renal disease
FBG	Fasting blood glucose
HbA1c	Glycated hemoglobin
HDL	High-density lipoprotein
HR	Hazard ratio
ICD-10	International Classification of Diseases, 10th Revision
IHD	Ischemic heart disease
IQR	Interquartile range
IR	Incidence rate
IRB	Institutional Review Board
KCD	Korean Standard Classification of Diseases
KCD-8	Korean Standard Classification of Diseases, 8th Revision
KNHIS	Korean National Health Insurance Service
LDL	Low-density lipoprotein
NHIS	National Health Insurance Service
NPDR	Non-proliferative diabetic retinopathy
PAD	Peripheral artery disease
PDR	Proliferative diabetic retinopathy
PSM	Propensity score matching
PY	Person-years
RRR	Relative risk ratio
SAS	Statistical Analysis System
SD	Standard deviation
SEP	Socioeconomic position
sHR	Subdistribution hazard ratio
SMD	Standardized mean difference
T2DM	Type 2 diabetes mellitus
VTDR	Vision-threatening diabetic retinopathy
