# Supplementary material for: Prior Cardiovascular Disease and Incident Diabetic Retinopathy in Newly Diagnosed Type 2 Diabetes Mellitus
Source: J Clin Med. 2026 Nov 3;15(21):8544. doi: 10.3390/jcm15218544 (PMC13764348; doi:10.3390/jcm15218544)
Supplement: Supplementary file 1 [file jcm-15-08544-s001.zip › jcm-4600439-supplementary.pdf]

Supplementary materials

Supplementary Figure S1. Flowchart of study participants selection

Supplementary Figure S2. Fine–Gray competing-risks cumulative incidence functions for DR outcomes.

Supplementary Figure S3. Five- and ten-year maximum DR severity distribution by prior CVD status

Supplementary Figure S4. Violin plot of time from NPDR to PDR (or VTDR) onset by prior CVD status

Supplementary Table S1. Detailed definitions of comorbidities and diabetes-related microvascular complications other than diabetic retinopathy

Supplementary Table S2. Clinical outcomes during follow-up according to prior CVD status

Supplementary Table S3. Time from NPDR to PDR (or VTDR) progression stratified by prior CVD status (N=2,727)

Supplementary Table S4. Sensitivity Analyses for the Association Between Prior CVD and Incident DR

Supplementary Figure S1. Flowchart of study participants selection

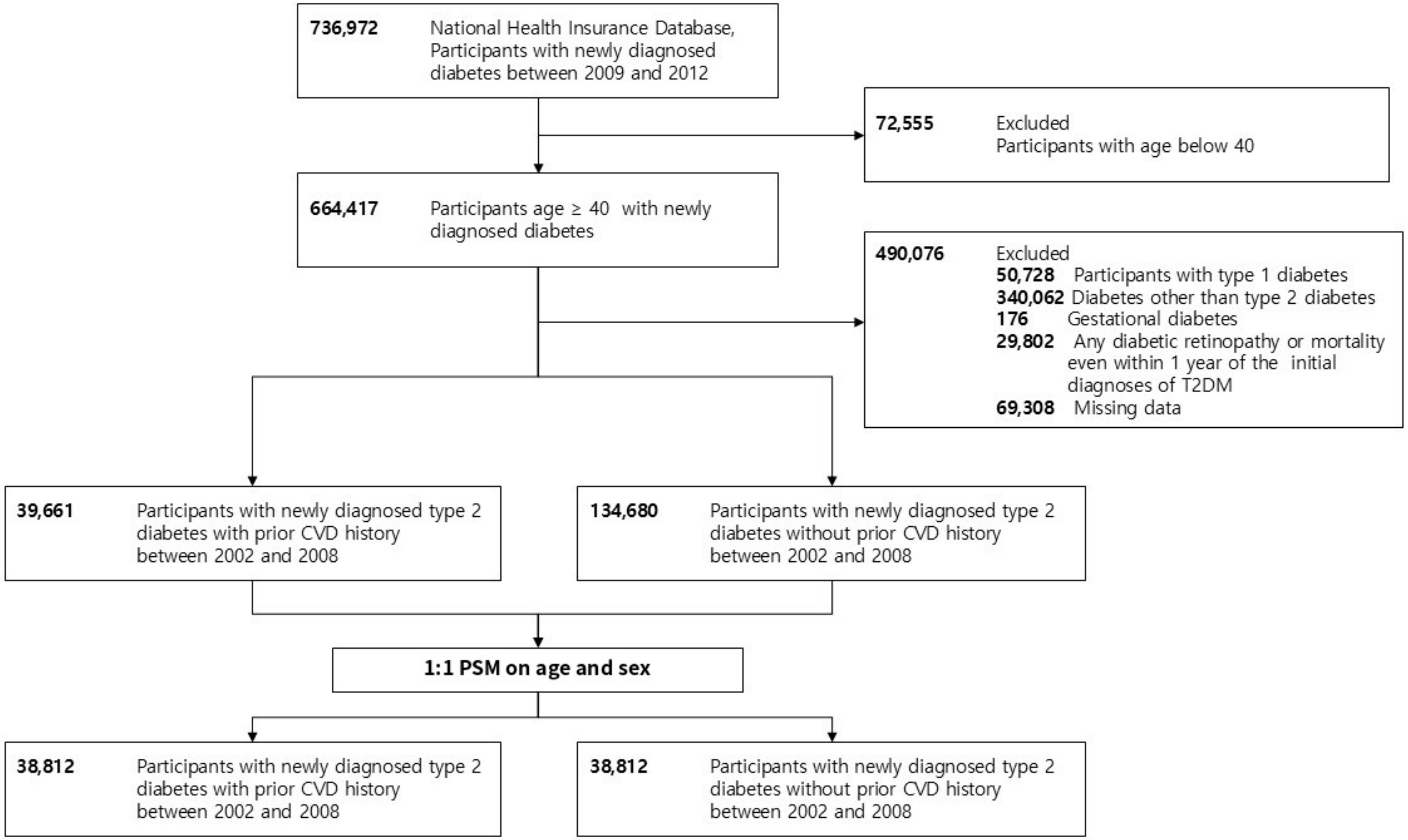

Of the 736,972 individuals newly diagnosed with diabetes between 2009 and 2012 in the KNHIS database, 72,555 were excluded because they were <40 years old. After further exclusion of patients with type 1 diabetes (n=50,728), other specified diabetes types (n=340,062), gestational diabetes (n=176), DR or death within 1 year of T2DM diagnosis (n=29,802), and missing health examination data (n=69,308), 174,341 patients remained. Of these, 39,661 had prior CVD between 2002 and 2008 and 134,680 did not. After 1:1 propensity score matching on age and sex, the final analytical cohort comprised 38,812 patients per group.

Abbreviations: CVD, cardiovascular disease; DR, diabetic retinopathy; KNHIS, Korean National Health Insurance Service; T2DM, type 2 diabetes mellitus.

Supplementary Figure S2. Fine–Gray competing-risks cumulative incidence functions for DR outcomes.

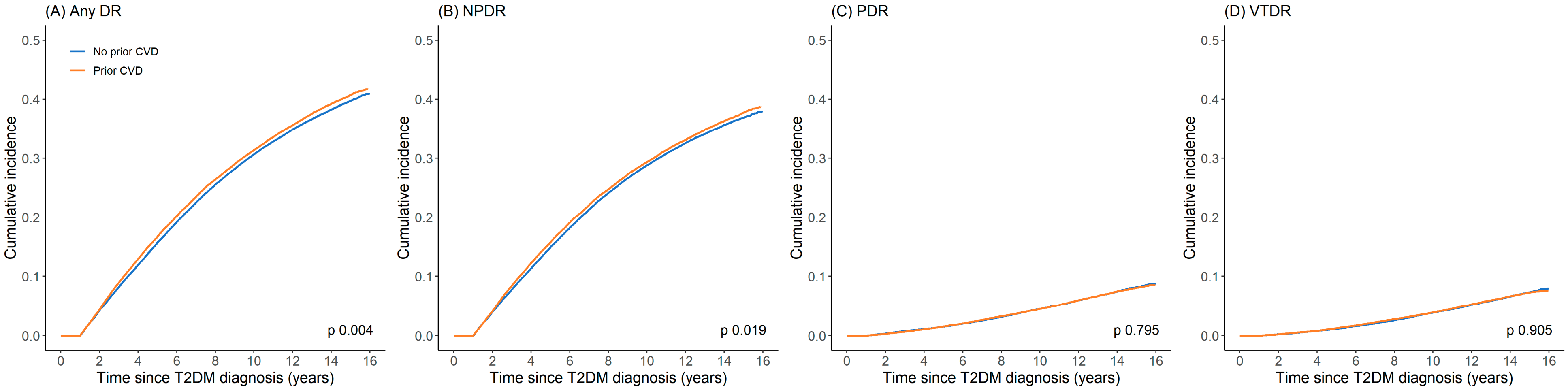

Cumulative incidence functions (CIFs) for (A) any DR, (B) NPDR, (C) PDR, and (D) VTDR estimated using the Fine–Gray method with all-cause death as the competing event in the propensity score–matched cohort. The orange lines represent the prior-CVD group, and the blue lines represent the no-prior-CVD group. The shaded regions indicate 95% confidence bands. Compared with the Kaplan–Meier curves in Figure 1, the between-group differences were attenuated in the CIFs, particularly for any DR and NPDR, which may partly reflect the higher competing mortality in the prior-CVD group (25.2% vs. 22.1%). P values (Gray's test) are shown in each panel.

CIF, cumulative incidence function; CVD, cardiovascular disease; DR, diabetic retinopathy; NPDR, nonproliferative diabetic retinopathy; PDR, proliferative diabetic retinopathy; VTDR, vision-threatening diabetic retinopathy.

Supplementary Figure S3. Five- and ten-year maximum DR severity distribution by prior CVD status

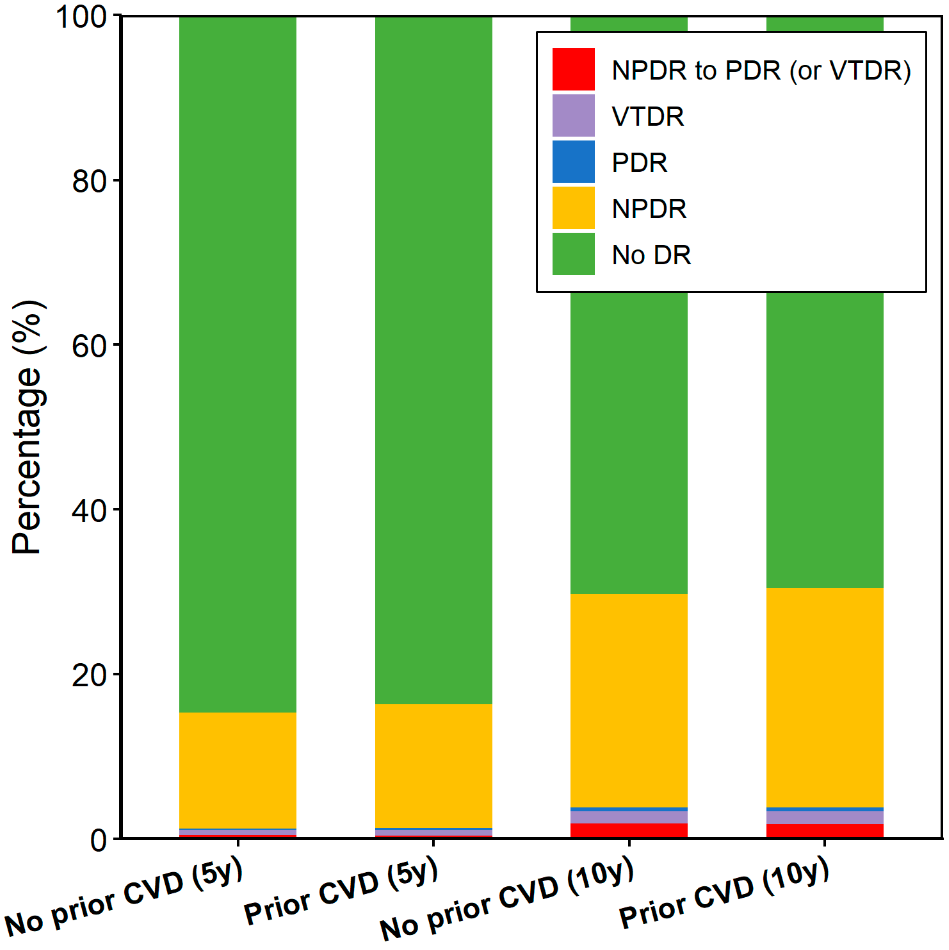

Stacked bar charts depicting the distribution of maximum observed DR severity: No DR (green), NPDR (yellow), NPDR-to-PDR (or VTDR) progression (red), de novo PDR (blue), and de novo VTDR (purple) at 5 and 10 years after T2DM diagnosis, stratified by prior CVD status. Proportions were based on a propensity score–matched cohort (n=38,812 per group).

Abbreviations: CVD, cardiovascular disease; DR, diabetic retinopathy; NPDR, nonproliferative diabetic retinopathy; PDR, proliferative diabetic retinopathy; VTDR, vision-threatening diabetic retinopathy; T2DM, type 2 diabetes mellitus.

Supplementary Figure S4. Violin plot of time from NPDR to PDR (or VTDR) onset by prior CVD status

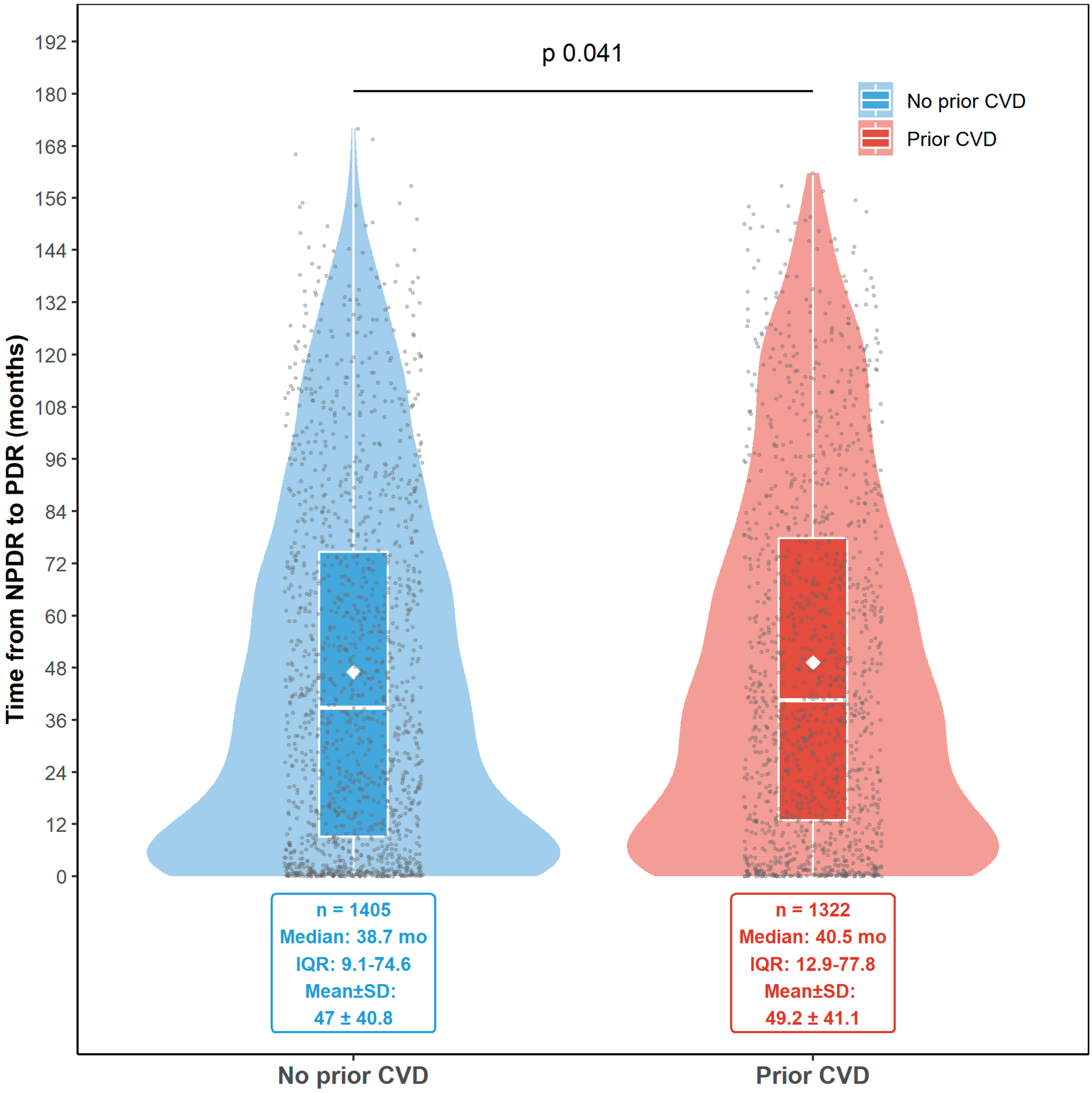

Distribution of time (months) from first NPDR diagnosis to first PDR (or VTDR) diagnosis in 2,727 patients who progressed to PDR or VTDR (no CVD, n=1,405; prior CVD, n=1,322). White diamonds indicate group means; horizontal white bars indicate medians; boxes represent IQRs. Individual data points are jittered. The prior-CVD group had a longer median time to PDR progression (40.5 months, IQR 12.9–77.8) than the no-CVD group (38.7 months, IQR 9.1–74.6; P=.041, Wilcoxon rank-sum test), suggesting that prior CVD was not associated with faster progression among patients who progressed.

Abbreviations: CVD, cardiovascular disease; IQR, interquartile range; NPDR, nonproliferative diabetic retinopathy; PDR, proliferative diabetic retinopathy; VTDR, vision-threatening diabetic retinopathy.

Supplementary Table S1. Detailed definitions of comorbidities and diabetes-related microvascular complications other than diabetic retinopathy

Variables	ICD-10 code
Comorbidities	
Hypertension	I10.1–I10.9
Dyslipidemia	E78.2, E78.4, or E78.5
Heart failure	I50
Chronic liver disease	K70-K77
Chronic obstructive pulmonary disease	J44
Diabetes-related vascular complications	
Diabetic nephropathy	E11.20, E11.21, E11.22, E11.28, N08.3, or N18
End-stage renal disease	N19, Z49, Z94.0, Z99.2 or O7011-7020, O7071-5, or R3280
Diabetic neuropathy	E11.40, E11.41, E11.42, E11.48, G59.0, G59.1, G63.2, G63.8, G99.0, or G99.1

All comorbidities were defined based on a full claims history (≥2 diagnoses at any time point).

Supplementary Table S2. Clinical outcomes during follow-up according to prior CVD status

Variable	Without Prior CVD (n=38,812)	With Prior CVD (n=38,812)	P value
Microvascular complications			
DR severity			.050
No DR	23,990 (61.8%)	23,652 (60.9%)	
NPDR	11,606 (29.9%)	11,961 (30.8%)	
PDR	644 (1.7%)	646 (1.7%)	
VTDR	2,572 (6.6%)	2,553 (6.6%)	
Diabetic nephropathy			<.001
None	28,994 (74.7%)	28,180 (72.6%)	
Without ESRD	8,072 (20.8%)	8,384 (21.6%)	
ESRD	1,746 (4.5%)	2,248 (5.8%)	
Diabetic neuropathy			<.001
None	25,598 (66.0%)	23,865 (61.5%)	
Yes	13,214 (34.1%)	14,947 (38.5%)	
Macrovascular complications (during follow-up)			
IHD	12,342 (31.8%)	20,301 (52.3%)	<.001
Stroke	12,735 (32.8%)	19,068 (49.1%)	<.001
PAD	21,359 (55.0%)	27,850 (71.8%)	<.001
All-cause mortality	8,567 (22.1%)	9,772 (25.2%)	<.001

Abbreviations: CVD, cardiovascular disease; DR, diabetic retinopathy; ESRD, end-stage renal disease; IHD, ischemic heart disease; NPDR, non-proliferative diabetic retinopathy; PAD, peripheral artery disease; PDR, proliferative diabetic retinopathy; VTDR, vision-threatening diabetic retinopathy.

Data are presented as n (%).

Supplementary Table S3. Time from NPDR to PDR (or VTDR) progression stratified by prior CVD status (N=2,727)

Variable	No Prior CVD (n=1,405)	Prior CVD (n=1,322)	P value
Time from NPDR to PDR (months)			.041
Median (IQR)	38.70 (9.10-74.63)	40.45 (12.87-77.80)	
Mean±SD	47.01±40.82	49.19±41.05	
Progression interval			
<1 year	390 (27.8%)	314 (23.8%)	
1–5 years	526 (37.4%)	541 (40.9%)	
>5 years	489 (34.8%)	467 (35.3%)	

The Wilcoxon rank-sum test was used for between-group comparisons of time to PDR progression.

Only patients who progressed from NPDR to PDR (or VTDR) were included in this analysis. The index date for this analysis was the date of first NPDR diagnosis.

Abbreviations: CVD, cardiovascular disease; IQR, interquartile range; NPDR, non-proliferative diabetic retinopathy; PDR, proliferative diabetic retinopathy; VTDR, vision-threatening diabetic retinopathy.

Supplementary Table S4. Sensitivity Analyses for the Association Between Prior CVD and Incident DR

Analysis	Population	Any DR HR (95% CI)	*P*-value	NPDR HR (95% CI)	*P*-value	PDR HR (95% CI)	*P*-value	VTDR HR (95% CI)	*P*-value
Main analysis	Total cohort (N=77,624)	1.01 (0.99-1.04)	0.262	1.01 (0.98-1.03)	0.605	1.01 (0.95-1.06)	0.835	1.00 (0.95-1.06)	0.893
Lag 2 years	Excluding DR within 2 years (N=74,278)	1.02 (0.99-1.04)	0.163	1.01 (0.98-1.04)	0.449	1.03 (0.97-1.09)	0.350	1.02 (0.96-1.09)	0.454
Ophthalmologist visit ≥1	Early exam only (N=28,494)	1.00 (0.97-1.04)	0.892	0.99 (0.96-1.03)	0.750	1.04 (0.96-1.13)	0.364	0.99 (0.91-1.08)	0.817
		Any DR Fine-Gray sHR (95% CI)	*P*-value	NPDR Fine-Gray sHR (95% CI)	*P*-value	PDR Fine-Gray sHR (95% CI)	*P*-value	VTDR Fine-Gray sHR (95% CI)	*P*-value
Main analysis	Total cohort (N=77,624)	0.97 (0.95-0.99)	0.014	0.97 (0.94-0.99)	0.005	0.96 (0.91-1.01)	0.108	0.95 (0.90-1.01)	0.089
Lag 2 years	Excluding DR within 2 years (N=74,278)	1.02 (0.99-1.04)	0.162	0.97 (0.94-0.99)	0.008	1.03 (0.97-1.09)	0.347	1.02 (0.96-1.09)	0.451
Ophthalmologist visit ≥1	Early exam only (N=28,494)	1.00 (0.97-1.04)	0.892	0.96 (0.92-1.00)	0.027	1.04 (0.96-1.13)	0.362	0.99 (0.91-1.08)	0.816

All models used fully adjusted Model 3 (age, sex, smoking, alcohol, insurance, residence, hypertension, dyslipidemia, BMI, fasting blood glucose, and pre-index ophthalmological visit). In the Fine–Gray models, all-cause death was treated as a competing event.

Abbreviations: BMI, body mass index; CVD, cardiovascular disease; DR, diabetic retinopathy; HR, hazard ratio; sHR, subdistribution hazard ratio; NPDR, nonproliferative diabetic retinopathy; PDR, proliferative diabetic retinopathy; VTDR, vision-threatening diabetic retinopathy.
